# Computational measures of irregularity molecular descriptors of octahedral and icosahedral networks

**DOI:** 10.3389/fchem.2024.1485184

**Published:** 2025-01-17

**Authors:** Xiujun Zhang, Hafiz Mutee ur Rehman, M. Mobeen Munir

**Affiliations:** ^1^ School of Computer Science, Chengdu University, Chengdu, China; ^2^ Department of Mathematics, Division of Science and Technology, University of Education Lahore, Lahore, Pakistan; ^3^ Department of Mathematics, University of the Punjab, Lahore, Pakistan

**Keywords:** irregularity indices, octahedral network, icosahedral network, computational comparisons, complexity

## Abstract

Irregularity measures tend to describe the complexity of networks. Chemical graph theory is a branch of mathematical chemistry that has a significant impact on the development of the chemical sciences. The study of irregularity indices has recently become one of the most active research areas in chemical graph theory. Irregularity indices help us to examine many chemical and biological properties of chemical structures under study. In this article, we study the irregularity indices of the octahedral and icosahedral networks. These networks are used in crystallography, where the topology and structural aspects are carrying some important facts to determine the properties of large structures theoretically. Our results play an important role in pharmacy, drug design, and many other applied areas. We also compared our results graphically to conclude the irregularity with a change in the parameter of structures.

## Introduction

Network structure and the pattern in networks carry important facts relating to the chemical properties. Because of the long molecular structure, the properties of some of these networks can’t be easily determined. Metallic-organic frameworks are such large networks whose symmetry and topology are incorporated in the bonding pattern and frequency of the atoms, (Jiang et al., 2016), (Zhao Y. et al., 2016) and (Liu et al., 2016). An indirect way of expressing the properties of these networks is through the use of topological index which fundamentally rely on the topological pattern of these networks.

One useful kind of topological index is the irregularity indices, which determine the complexity and degree of irregular patterns in the networks and graphs. These networks or graphs can be representative models of some crystallographic structures or a polymer where lines represent bonding patterns and vertices show atoms. Irregularity indices of fairly large chemical structures, such as metal organic frameworks, are important not only for characterization of structures but also for computing their physico-chemical properties, which have been otherwise rather difficult to compute for such large networks of importance in chemistry. In some of these networks, covalent fibers are weaved into crystals, which why these networks are becoming increasingly interesting in recent years.

Chemical graph theory is a thriving field with rich applications in industry and pharmacy. Graphs are models of physical networks described by two main sets of entities named as edges and vertices. The number of edges incident to each vertex is termed the degree of vertex. According to (Gutman and Polansky, 1986), a network or graph is considered regular if every vertex has the same degree. But until famous mathematician Paul Erdös stressed this in the study of irregular graphs for the first time in history, irregular graphs were unable to draw in the audience (Majcher and Michael, 1997). The greatest size of irregularity in a network, as suggested by Collatz and Sinogowitz (1957), was the subject of an open topic that he presented. Subsequently, irregularity became so widely accepted that a new class of topological indices emerged, which are now called irregularity indices. The disparity of complex systems can be predicted by these metrics. These systems have a number of well-known topological characteristics, including self-similarity, scale-freeness, network motifs, and small-worldness (Albert et al., 2000). In summary, there is a stark difference between the power-law degree distribution of complex networks and the regularity found in random models such as the one put forth by Erdös and Rényi (1960).

An irregularity index is defined as a topological invariant that vanishes for a regular graph but is non-zero for a non-regular graph. Erdös declared, “The determination of extreme size of highly irregular graphs of given order” to be an unresolved subject at the Second Krakow Conference on Graph Theory (1994) (Chartrand et al., 1988). Since then, irregular graphs and their degree of irregularity have emerged as one of graph theory’s most fundamental open problems. A graph is said to be perfect if each vertex has a different degree than the others. The writers of (Behzad and Chartrand, 1947) established that no graph is flawless. The graphs in between are known as quasi-perfect graphs, because all but two vertices have distinct degrees (Majcher and Michael, 1997). Indices are simplified ways of expressing anomalies, (Horoldagva et al., 2016; Liu et al., 2014), conducted unique research on these irregularity indices. The first such irregularity index was established by Collatz and Sinogowitz (1957). Most of these indices utilize the concept of edge imbalance, defined as *imball*_*uv*_ = |*d*_u_–*d*_v_|, (Dorogovtsev and Mendes, 2000; Krapivsky et al., 2000). The Albertson index, AL(G), was introduced by Albertson in (Albertson, 1997) and is defined as *AL*(*G*) = ^∑^_uv∈*E*_|*d*_*u*_ − *d*_*v*_|. The irregularity indices IRL(G) and IRLU(G) were introduced by Vukicevic and Gasparov, (Albert et al., 2000), and are defined as $\text{IRL}\left(G\right)=\sum_{uv\in E}\left|\ln d_{u}-\ln d_{v}\right|$ , and $\text{IRLU}\left(G\right)=\sum_{uv\in E}\frac{\left|d_{u}-d_{v}\right|}{\min\left(d_{u},d_{v}\right)}$ . Recently, (Abdo et al., 2014), introduced a new concept called the “total irregularity measure of a graph G,” defined as $R_{t}\left(G\right)=\frac{1}{2}\sum_{uv\in E}\left|d_{u}-d_{v}\right|$ , (Erdös and Rényi, 1960; Estrada et al., 1998; Reti et al., 2018). Recently, Gutman et al. introduced the IRF(G) irregularity index of the graph G, defined as *IRF* ^(^ *G* ^)^ = ^∑^_uv∈*E*_(*d*_*u*_ − *d*_*v*_)^2^ (Bell, 1992). The Randić index is closely connected to an irregularity measure, defined as $\left(G\right)=\sum_{uv\in E}{\left(d_{u}^{-1/2}-d_{v}^{-1/2}\right)}^{2}$ , (Albertson, 1997). Further details on similar irregularity indices may be found in (Abdo and Dimitrov, 2014a). These indices are defined as follows: $IRDIF\left(G\right)=\sum_{uv\in E}\left|\frac{d_{u}}{d_{v}}-\frac{d_{v}}{d_{u}}\right|$ , $IRLF\left(G\right)=\sum_{uv\in E\;}\frac{\left|d_{u}-d_{v}\right|}{\sqrt{d_{u}d_{v}}}$ , $IR\text{LA}\left(G\right)=2\sum_{uv\in E}\frac{\left|d_{u}-d_{v}\right|}{\left(d_{u}+d_{v}\right)}$ , $IRD1\left(G\right)=\sum_{uv\in E}\ln\;\left(1+\left|d_{u}-d_{v}\right|\right)$ , $IRGA\left(G\right)=2\sum_{uv\in E}\ln\frac{d_{u}+d_{v}}{2\sqrt{d_{u}d_{v}}}$ , and $IRD\left(G\right)=\sum_{uv\in E}{\left(d_{u}^{1/2}-d_{v}^{1/2}\right)}^{2}$ . More information can be found in (Abdo and Dimitrov, 2014b; Gutman, 2018; Hu et al., 2005; Li and Gutman, 2006). Recently, (Zahid et al., 2019), calculated the irregularity indices for nanotubes. (Gao et al., 2017; Gao et al., 2019). examined irregularity measurements for different dendrimer architectures (Hussain et al., 2019a; Hussain et al., 2019b). Estimated the irregularity indices for benzenoid systems, nanostar dendrimers, and boron nanotubes. Furthermore, (Xie et al., 2019), estimated these indices for fullerenes and polymer dendrimers. Quiet recently, many new topological characterizations of several chemical structures based on topological indices have been presented along with various applications, (Zhang et al., 2024; Prabhu et al., 2023; Prabhu et al., 2024; Govardhan et al., 2023; Saravanan et al., 2022).

**FIGURE 1:**
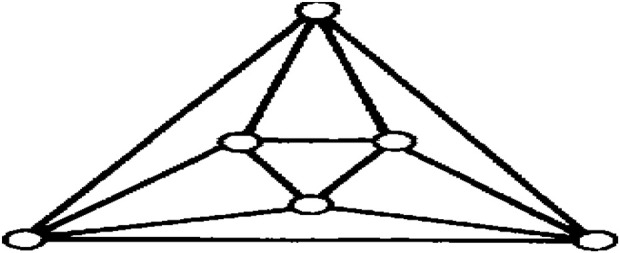
Structure of octahedron.

**FIGURE 2:**
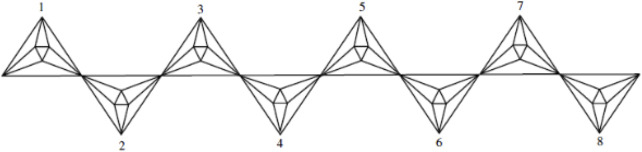
Chain octahedral structure.

**FIGURE 3:**
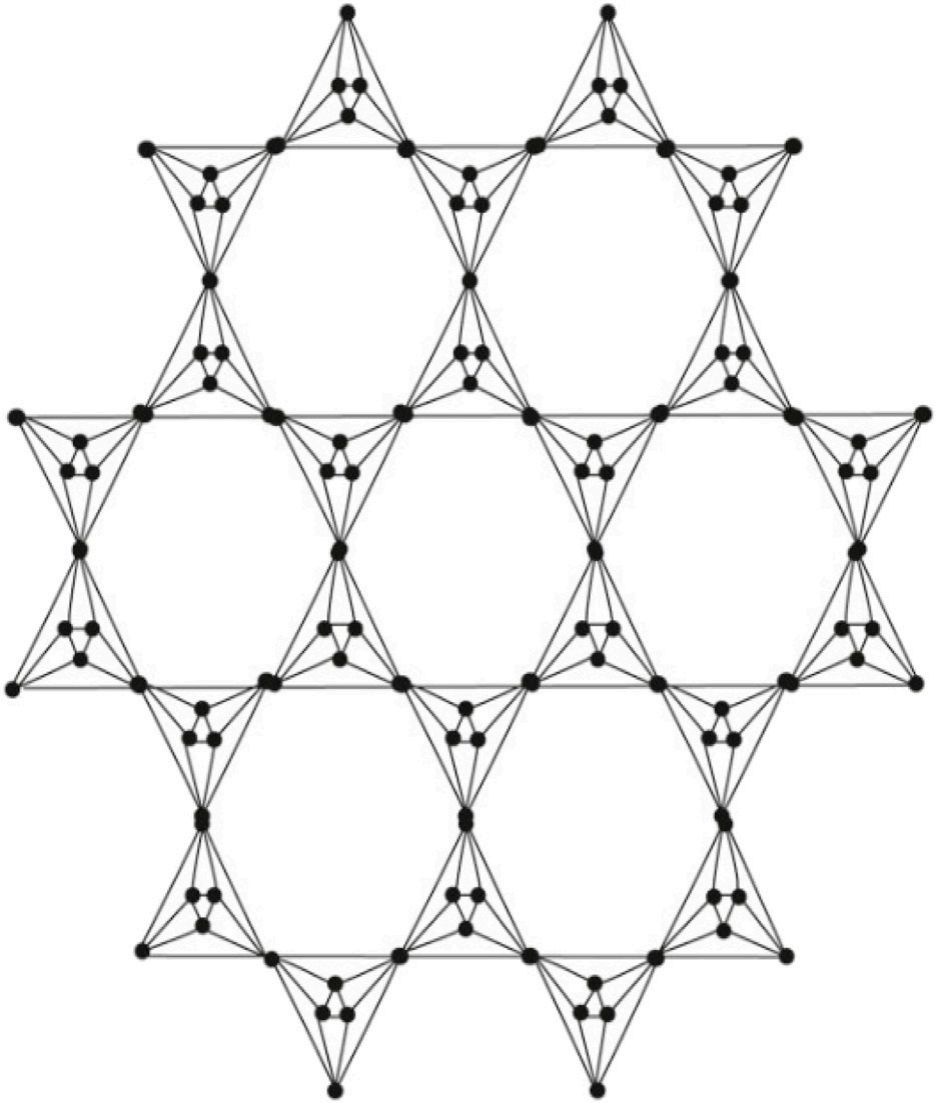
Octahedral network.

**FIGURE 4:**
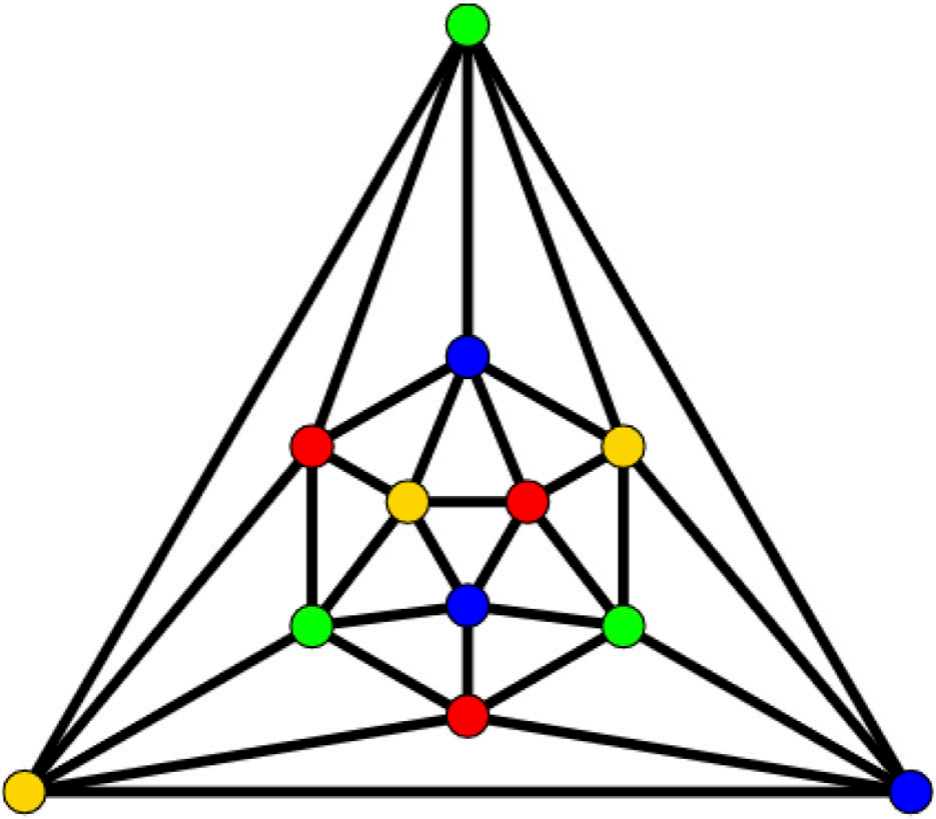
Structure of icosahedron.

**FIGURE 5:**
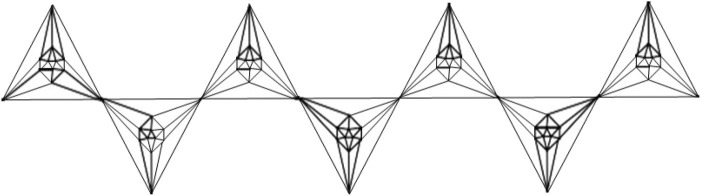
Structure of chain icosahedron.

**FIGURE 6:**
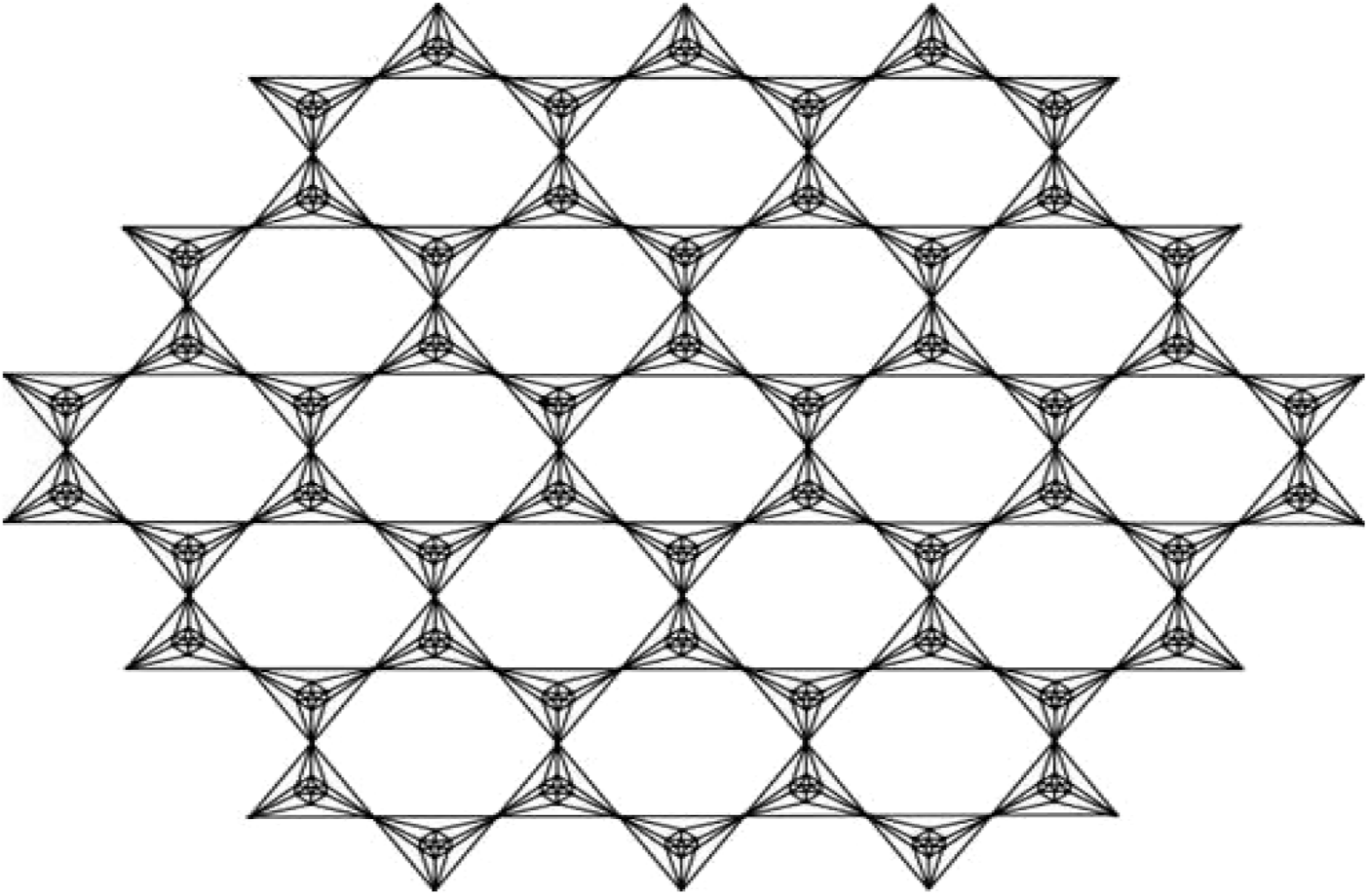
Icosahedral network.

This article examines the irregularity of well-known chemical networks by computing the irregularity indices for octahedral and icosahedral networks. Our goal is to determine which of these networks exhibits greater irregularity. Specifically, we evaluate the degree of irregularity in the octahedral network *OTn* and the icosahedral network *ISn*. Figures 1–3 depict the molecular graphs of the octahedral networks, while Figures 4–6 illustrate the molecular graphs of the icosahedral networks. The motivation for this study stems from previous findings that irregularity indices can closely approximate properties such as entropy, standard enthalpy, vaporization, and acentric factors of octane isomers (Abdo et al., 2014). These figures display the molecular patterns and topologies of the two networks under investigation.

## Octahedral networks *OT*_*n*_

An octahedron graph, shown in Figure 1, is a polyhedral graph corresponding to the skeleton of a platonic solid. This platonic graph consists of 6 vertices and 12 edges. The analogs of this structure play a vital role in the fields of reticular chemistry, which deals with the synthesis and properties of metal-organic frameworks. The different types of octahedral structures arise from the ways these octahedra can be connected. A chain octahedral structure of dimension n denoted as *CHO*_*n*_ is obtained by arranging *n* octahedra linearly as shown in Figure 2. The number of vertices and edges of *CHO*_*n*_ are 5*n* + 1 and 12*n*, respectively. An octahedral sheet-like structure is a ring of octahedral structures that are linked to other rings by sharing corner vertices in a two-dimensional plane. An octahedral network of dimension *n* is denoted by *OT*_*n*_, where *n* is the order of circumscribing, as shown in Figure 3, The number of vertices and edges in *OT*_*n*_ with *n* ≥ 1 are 27*n* ^2^ + 3*n* and 72*n* ^2^, respectively.

### Icosahedral networks *ISn*

An icosahedron graph is also a platonic graph, having 12 vertices and 30 edges, as shown in Figure 4. The analogs of the frameworks considered here are also the backbones of recent materials of reticular chemistry. The chain icosahedral framework of dimension n is denoted by *CHI*_*n*_ and is shown in Figure 5. It has 11*n* + 1 number of vertices and 30*n* number of edges. The icosahedral network is obtained from the octahedral network by replacing all the octahedra with the icosahedra. An *n*-dimensional icosahedral network is denoted by *IS*_*n*_ is shown in Figure 6. It has 63*n* ^2^ + 3*n* number of vertices and 180*n* ^2^ number of edges. We discuss the irregularity indices of this network as follows.

## Main results

In this section, we present our main theoretical results. Here we denote *Γ* = *OT*_*n*_ be the octahedral network then.

### Theorem 1

*Let* (Γ, *x*, *y*) *be the graph of the* octahedral network*s OT*_n_ *, then the irregularity indices of* (Γ, *x*, *y*) *are*.

1. *IRDI*(Γ, *x*, *y*) = 54*n* ^2^
2. *A*(Γ, *x*, *y*) = 144*n* ^2^
3. *IR*(Γ, *x*, *y*) = 24.953299*n* ^2^
4. *IRL*(Γ, *x*, *y*) = 36*n* ^2^
5. *IRL*(Γ, *x*, *y*) = 25.455844*n* ^2^
6. *IR*(Γ, *x*, *y*) = 576*n* ^2^
7. *IRL*(Γ, *x*, *y*) = 24*n* ^2^
8. *IRD1* = 57.939768*n* ^2^
9. *IR*(Γ, *x*, *y*) = 0.772078*n* ^2^
10. *IRG*(Γ, *x*, *y*) = 4.240189*n* ^2^
11. *IR*(Γ, *x*, *y*) = 24.70649*n* ^2^
12. *IRR*(Γ, *x*, *y*) = 72*n* ^2^

In order to prove the above theorem, we have to consider Figure 3. We can see that the edges of (Γ, *x*, *y*) admit the following partition in Table 1.

**TABLE 1:** Edge partition of Octahedral network *OT*_n_.

Number of edges (*d*_*u*_, *d*_*v*_)	Number of indices
(4, 4)	18*n* ^2^ + 12*n*
(4, 8)	36*n* ^2^
(8, 8)	18*n* ^2^ − 12*n*

Now using Table 1 and the above definitions, we have:

1. $IRDIF\left(G\right)=\sum_{uv\in E}\left|\frac{d_{u}}{d_{v}}-\frac{d_{v}}{d_{u}}\right|$
$$IRDIF\left(\Gamma ,x,y\right)=\left(18n^{2}+12n\right)\left|\frac{4}{4}-\frac{4}{4}\right|+36n^{2}\left|\frac{8}{4}-\frac{4}{8}\right|+\left(18n^{2}-12n\right)\left|\frac{8}{8}-\frac{8}{8}\right|=54n^{2}$$
2. *A*(*G*) = ^∑^_uv∈*E*_|*d*_*u*_ − *d*_*v*_|
$$A\left(\Gamma ,x,y\right)=\left(18n^{2}+12n\right)\left|4\begin{array}{cc} - & 4 \end{array}\right|+36n^{2}\left|8\begin{array}{cc} - & 4 \end{array}\right|+\left(18n^{2}-12n\right)\left|8\begin{array}{cc} - & 8 \end{array}\right|=144n^{2}.$$
3. *IR*(*G*) = ^∑^_uv∈*E*_|*lnd*_*u*_ − *lnd*_*v*_|
$$\text{IRL}\left(\Gamma ,x,y\right)=\left(18n^{2}+12n\right)\left|\ln4-\ln4\right|+36n^{2}\left|\ln8-\ln4\right|+\left(18n^{2}-12n\right)\left|\ln8-\ln8\right|=36n^{2}\ln2=24.953299n^{2}$$
4. $\text{IRLU}\left(G\right)=\sum_{uv\in E}\frac{\left|d_{u}-d_{v}\right|}{\min\left(d_{u},d_{v}\right)}$
$$\text{IRLU}\left(\Gamma ,x,y\right)=\left(18n^{2}+12n\right)\frac{\left|4-4\right|}{4}+36n^{2}\frac{\left|8-4\right|}{4}+\left(18n^{2}-12n\right)\frac{\left|8-8\right|}{8}=36n^{2}$$
5. $IRLF\left(G\right)=\sum_{uv\in E\;}\frac{\left|d_{u}-d_{v}\right|}{\sqrt{d_{u}d_{v}}}$ ,
$$IRLF\left(\Gamma ,x,y\right)=\left(18n^{2}+12n\right)\;\frac{\left|4-4\right|}{\sqrt{4\times 4}}+36n^{2}\frac{\left|8-4\right|}{\sqrt{8\times 4}}+\left(18n^{2}-12n\right)\frac{\left|8-8\right|}{\sqrt{8\times 8}}=25.455844n^{2}$$
6. *IR*(*G*) = ^∑^_uv∈*E*_(*d*_*u*_ − *d*_*v*_)^2^
$$IR\left(\Gamma ,x,y\right)=\left(18n^{2}+12n\right){\left(4-4\right)}^{2}+36n^{2}{\left(8-4\right)}^{2}+\left(18n^{2}-12n\right){\left(8-8\right)}^{2}=576n^{2}.$$
7. $IR\text{LA}\left(G\right)=2\sum_{uv\in E}\frac{\left|d_{u}-d_{v}\right|}{\left(d_{u}+d_{v}\right)}$
$$IR\text{LA}\left(\Gamma ,x,y\right)=2\left[\left(18n^{2}+12n\right)\frac{\left|4-4\right|}{4+4}+36n^{2}\frac{\left|8-4\right|}{8+4}+\left(18n^{2}-12n\right)\frac{\left|8-8\right|}{8+8}\right]=24n^{2}$$
8. $IRD1\left(G\right)=\sum_{uv\in E}\ln\;\left(1+\left|d_{u}-d_{v}\right|\right)$
$$IRD1\left(\Gamma ,x,y\right)=\left(18n^{2}+12n\right)\ln\;\left(1+\left|4-4\right|\right)+36n^{2}\ln\;\left(1+\left|8-4\right|\right)+\left(18n^{2}-12n\right)\ln\;\left(1+\left|8-8\right|\right)=36n^{2}\ln5=57.9397648n^{2}$$
9. $IRA\left(G\right)=\sum_{uv\in E}{\left(d_{u}^{-1/2}-d_{v}^{-1/2}\right)}^{2}$
$$IRA\left(\Gamma ,x,y\right)=\left(18n^{2}+12n\right){\left(4^{-0.5}-4^{-0.5}\right)}^{2}+36n^{2}{\left(8^{-0.5}-4^{-0.5}\right)}^{2}+\left(18n^{2}-12n\right){\left(8^{-0.5}-8^{-0.5}\right)}^{2}=0.772078n^{2}$$
10. $IRGA\left(G\right)=2\sum_{uv\in E}\ln\frac{d_{u}+d_{v}}{2\sqrt{d_{u}d_{v}}}$
$$IRGA\left(\Gamma ,x,y\right)=2\left[\left(18n^{2}+12n\right)\ln\frac{4+4}{2\sqrt{4\times 4}}+36n^{2}\ln\frac{8+4}{2\sqrt{8\times 4}}+\left(18n^{2}-12n\right)\ln\frac{8+8}{2\sqrt{8\times 8}}\right]=4.240189\;n^{2}$$
11. $IRB\left(G\right)=\sum_{uv\in E}\left({d_{u}}^{1/2}-{d_{v}}^{1/2}\right)$ ^2^
$$IRB\left(\Gamma ,x,y\right)=\left(18n^{2}+12n\right){\left(4^{1/2}-4^{1/2}\right)}^{2}+36n^{2}{\left(8^{1/2}-4^{1/2}\right)}^{2}+\left(18n^{2}-12n\right){\left(8^{1/2}-8^{1/2}\right)}^{2}=24.70649n^{2}$$
12. $IRR_{t}\left(G\right)=\frac{1}{2}\sum_{uv\in E}\left|d_{u}-d_{v}\right|$
$$IRR_{t}\left(\Gamma ,x,y\right)=\frac{1}{2}\left[\left(18n^{2}+12n\right)\left|4-4\right|+36n^{2}\left|8-4\right|+\left(18n^{2}-12n\right)\left|8-8\right|\right]=72n^{2}$$

Table 2 shows the values of these irregularity indices for some test values of parameter n.

**TABLE 2:** Irregularity indices for Octahedral network *OT*_*n*_.

Irregularity indices	n = 1	n = 2	n = 3	n = 4	n = 5
$IRDIF\left(G\right)=\sum_{uv\in E}\left|\frac{d_{u}}{d_{v}}-\frac{d_{v}}{d_{u}}\right|$	54	216	486	864	1,350
*AL*(G) = ∑_*uv∈E*_|*d*_*u*_ − *d*_*v*_|	144	576	1,296	2,304	3,600
*IRL*(G) = ∑_*uv∈E*_|*lnd*_*u*_ − *lnd*_*v*_|	24.953299	99.813196	224.579691	399.252784	623.832475
$\text{IRLU}\left(G\right)=\sum_{uv\in E}\frac{\left|d_{u}-d_{v}\right|}{\min\left(d_{u},d_{v}\right)}$	36	144	324	576	900
$\text{IRLF}\left(G\right)=\sum_{uv\in E}\frac{\left|d_{u}-d_{v}\right|}{\sqrt{d_{u}d_{v}}}$	25.455844	101.823376	229.102596	407.293504	636.3961
*IRF*(G) = ∑_*uv∈E*_(*d*_*u*_ − *d*_*v*_)^2^	576	2,304	5,184	9,216	14,400
$IR\text{LA}\left(G\right)=2\sum_{uv\in E}\frac{\left|d_{u}-d_{v}\right|}{\left(d_{u}+d_{v}\right)}$	24	96	216	384	600
*IRD1* = ∑_*uv∈E*_ *ln*{1 + |*d*_*v*_ − *d*_*v*_|}	57.939768	231.759072	521.457912	927.036288	1,448.494200
$IRA\left(G\right)=\sum_{uv\in E}{\left(d_{u}^{-1/2}-d_{v}^{-1/2}\right)}^{2}$	0.772078	3.088312	6.948702	12.353248	19.30195
$\text{IRGA}\left(G\right)=2\sum_{uv\in E}\ln\frac{d_{u}+d_{v}}{2\sqrt{d_{u}d_{v}}}$	4.240189	16.960756	38.161701	67.843024	106.004725
$\text{IRB}\left(G\right)=\sum_{uv\in E}{\left({d_{u}}^{1/2}-{d_{v}}^{1/2}\right)}^{2}$	24.70649	98.82596	222.35841	395.30384	617.66225
$IRR_{t}\left(G\right)=\frac{1}{2}\sum_{uv\in E}\left|d_{u}-d_{v}\right|$	72	288	648	1,152	1800

### Theorem 2

Let *IS*_*n*_ be the Icosahedral network, which we denote by Γ. The irregularity indices of Γ are as follows:

1. *IRDI*(Γ, *x*, *y*) = 81*n* ^2^ − 9*n*
2. *A*(Γ, *x*, *y*) = 270*n* ^2^ − 30*n*
3. *IR*(Γ, *x*, *y*) = 37.429938*n* ^2^ − 4.158883*n*
4. *IRL* ^(^Γ, *x*, *y* ^)^ = 54*n* ^2^ − 6*n*
5. *IRL*(Γ, *x*, *y*) = 38.183778*n* ^2^ − 4.242641*n*
6. *IR*(Γ, *x*, *y*) = 1350*n* ^2^ − 150*n*
7. *IRL*(Γ, *x*, *y*) = $36n^{2}-4n$

8. *IRD*1 = $96.7550\;n^{2}\;‐\;10.75056\;n$

9. *IR*(Γ, *x*, *y*) = $0.926494n^{2}-0.102944n$

10. *IRG*(Γ, *x*, *y*) = 114.5513 n^2 - 12.72792 n
11. *IR*(Γ, *x*, *y*) = $46.32468n^{2}-5.14719n$

12. *IRR*(Γ, *x*, *y*) = 135*n* ^2^ − 15*n*

To prove the above theorem, we must consider Figure 6. As shown, the edges admit the following partition, presented in Table 3.

**TABLE 3:** Edge partition of Icosahedral network *IS*_*n*_.

Number of edges (*d*_*u*_, *d*_*v*_)	Number of indices
(5, 5)	108*n* ^2^ + 18*n*
(5,10)	54*n* ^2^ − 6*n*
(10, 10)	18*n* ^2^ − 12*n*

Now using Table 3 and the above definitions, we have:

1. $IRDIF\left(G\right)=\sum_{uv\in E}\left|\frac{d_{u}}{d_{v}}-\frac{d_{v}}{d_{u}}\right|$
$$IRDIF\left(\Gamma ,x,y\right)=\left(108n^{2}+18n\right)\left|\frac{5}{5}-\frac{5}{5}\right|+\left(54n^{2}-6n\right)\left|\frac{10}{5}-\frac{5}{10}\right|+\left(18n^{2}-12n\right)\left|\frac{10}{10}-\frac{10}{10}\right|=81n^{2}-9n.$$
2. *A*(*G*) = ^∑^_uv∈*E*_|*d*_*u*_ − *d*_*v*_|
$$A\left(\Gamma ,x,y\right)=\left(108n^{2}+18n\right)\left|5-5\right|+\left(54n^{2}-6n\right)\left|10-5\right|+\left(18n^{2}-12n\right)\left|10-10\right|=270n^{2}-30n.$$
3. IRL (*G*) = ^∑^_uv∈*E*_|*lnd*_*u*_ − *lnd*_*v*_|
$$IRL\left(\Gamma ,x,y\right)=\left(108n^{2}+18n\right)\left|\ln5-\ln5\right|+\left(54n^{2}-6n\right)\left|\ln10-\ln\;5\right|+\left(18n^{2}-12n\right)\left|\ln10-\ln10\right|=37.429938n^{2}-4.158883n.$$
4. $IRLU\left(G\right)=\sum_{uv\in E}\frac{\left|d_{u}-d_{v}\right|}{\min\left(d_{u},d_{v}\right)}$
$$IRLU\left(\Gamma ,x,y\right)=\left(108n^{2}+18n\right)\frac{\left|5-5\right|}{5}+\left(54n^{2}-6n\right)\frac{\left|10-5\right|}{5}+\left(18n^{2}-12n\right)\frac{\left|10-10\right|}{10}=54n^{2}-6n.$$
5. $IRLF\left(G\right)=\sum_{uv\in E}\frac{\left|d_{u}-d_{v}\right|}{\sqrt{d_{u}d_{v}}}$
$$IRLF\left(\Gamma ,x,y\right)=\left(108n^{2}+18n\right)\frac{\left|5-5\right|}{\sqrt{5\times 5}}+\left(\left(54n^{2}-6n\right)\right)\frac{\left|10-5\right|}{\sqrt{10\times 5}}+\left(18n^{2}-12n\right)\frac{\left|10-10\right|}{\sqrt{10\times 10}}=38.18377\;n^{2}‐4.242641\;n.$$
6. *IR*(*G*) = ^∑^_uv∈*E*_(*d*_*u*_ − *d*_*v*_)^2^
$$IR\left(\Gamma ,x,y\right)=\left(108n^{2}+18n\right){\left(5-5\right)}^{2}+\left(54n^{2}-6n\right){\left(10-5\right)}^{2}+\left(18n^{2}-12n\right){\left(10-10\right)}^{2}=1350n^{2}-150n.$$
7. $IR\text{LA}\left(G\right)=2\sum_{uv\in E}\frac{\left|d_{u}-d_{v}\right|}{\left(d_{u}+d_{v}\right)}$
$$IR\text{LA}\left(\Gamma ,x,y\right)=2\left[\left(108n^{2}+18n\right)\frac{\left|5-5\right|}{5+5}+\left(54n^{2}-6n\right)\frac{\left|10-5\right|}{10+5}+\left(18n^{2}-12n\right)\frac{\left|10-10\right|}{10+10}\right]=36n^{2}-4n.$$
8. $\text{IRD}1\left(G\right)=\sum_{uv\in E}\ln\left(1+\left|d_{u}-d_{v}\right|\right)$
$$\text{IRD}1\left(\Gamma ,x,y\right)=\left(108n^{2}+18n\right)\ln\left(1+\left|5-5\right|\right)+\left(54n^{2}-6n\right)\ln\left(1+\left|10-5\right|\right)+\left(18n^{2}-12n\right)\ln\left(1+\left|10-10\right|\right)=\left(54n^{2}-6n\right)\ln6=96.7550\;n^{2}\;‐\;10.75056\;n.$$
9. $IRA\left(G\right)=\sum_{uv\in E}{\left(d_{u}^{-1/2}-d_{v}^{-1/2}\right)}^{2}$
$$IRA\left(\Gamma ,x,y\right)=\left(108n^{2}+18n\right){\left(5^{-0.5}-5\right)}^{2}+\left(54n^{2}-6n\right){\left(10^{-0.5}-5^{-0.5}\right)}^{2}+\left(18n^{2}-12n\right){\left(10^{-0.5}-10^{-0.5}\right)}^{2}=0.926494n^{2}-0.102944n.$$
10. $\text{IRGA}\left(G\right)=2\sum_{uv\in E}\ln\frac{d_{u}+d_{v}}{2\sqrt{d_{u}d_{v}}}$
$$\text{IRGA}\left(\Gamma ,x,y\right)=2\left[\left(108n^{2}+18n\right)\ln\left(\frac{5+5}{2\sqrt{5\times 5}}\right)+\left(54n^{2}-6n\right)\ln\left(\frac{10+5}{2\sqrt{10\times 5}}\right)+\left(18n^{2}-12n\right)\ln\left(\frac{10+10}{2\sqrt{10\times 10}}\right)\right]=4.240189\;n^{2}.$$
11. $\text{IRB}\left(G\right)=\sum_{uv\in E}{\left({d_{u}}^{1/2}-{d_{v}}^{1/2}\right)}^{2}$
$$\text{IRB}\left(\Gamma ,x,y\right)=\left(108n^{2}+18n\right){\left(5^{1/2}-5^{1/2}\right)}^{2}+\left(54n^{2}-6n\right){\left(10^{1/2}-5^{1/2}\right)}^{2}+\left(18n^{2}-12n\right){\left(10^{1/2}-10^{1/2}\right)}^{2}=46.32468n^{2}-5.14719n.$$
12. $IRR_{t}\left(G\right)=\frac{1}{2}\sum_{uv\in E}\left|d_{u}-d_{v}\right|$
$$IRR_{t}\left(\Gamma ,x,y\right)=\frac{1}{2}\left[\left(108n^{2}+18n\right)\left|5-5\right|+\left(54n^{2}-6n\right)\left|10-5\right|+\left(18n^{2}-12n\right)\left|10-10\right|\right]=135n^{2}-15n.$$

Table 4 shows the values of these irregularity indices for some test values of parameter n.

**TABLE 4:** Irregularity indices for Isosahedral network *IS*_*n*_.

Irregularity indices	n = 1	n = 2	n = 3	n = 4	n = 5
$IRDIF\left(G\right)=\sum_{uv\in E}\left|\frac{d_{u}}{d_{v}}-\frac{d_{v}}{d_{u}}\right|$	72	306	702	1,260	1,980
*AL*(G) = ∑_*uv∈E*_|*d*_*u*_ − *d*_*v*_|	240	1,020	2,340	4,200	6,600
*IRL*(G) = ∑_*uv∈E*_|*lnd*_*u*_ − *lnd*_*v*_|	33.271056	141.40198	324.392796	582.24348	914.954040
$\text{IRLU}\left(G\right)=\sum_{uv\in E}\frac{\left|d_{u}-d_{v}\right|}{\min\left(d_{u},d_{v}\right)}$	48	204	468	840	1,320
$\text{IRLF}\left(G\right)=\sum_{uv\in E}\frac{\left|d_{u}-d_{v}\right|}{\sqrt{d_{u}d_{v}}}$	33.941136	144.249828	330.926076	593.969880	933.381240
*IRF*(G) = ∑_*uv∈E*_(*d*_*u*_ − *d*_*v*_)^2^	1,200	5,100	11,700	21,000	33,000
$IR\text{LA}\left(G\right)=2\sum_{uv\in E}\frac{\left|d_{u}-d_{v}\right|}{\left(d_{u}+d_{v}\right)}$	32.000016	136.000068	312.000156	560.000280	880.000440
*IRD1* = ∑_*uv∈E*_ *ln*{1 + |*d*_*v*_ − *d*_*v*_|}	86.004432	365.518836	838.543212	1,505.07756	2,365.1218
$IRA\left(G\right)=\sum_{uv\in E}{\left(d_{u}^{-1/2}-d_{v}^{-1/2}\right)}^{2}$	0.823536	3.500028	8.029476	14.411880	22.647240
$\text{IRGA}\left(G\right)=2\sum_{uv\in E}\ln\frac{d_{u}+d_{v}}{2\sqrt{d_{u}d_{v}}}$	2.826768	12.013764	27.560988	49.468440	77.736120
$\text{IRB}\left(G\right)=\sum_{uv\in E}{\left({d_{u}}^{1/2}-{d_{v}}^{1/2}\right)}^{2}$	41.177472	175.004256	401.480352	720.736120	1,132.380480
$IRR_{t}\left(G\right)=\frac{1}{2}\sum_{uv\in E}\left|d_{u}-d_{v}\right|$	120	510	1,170	2,100	3,300

## Graphical analysis, discussions and conclusion

**FIGURE 7:**
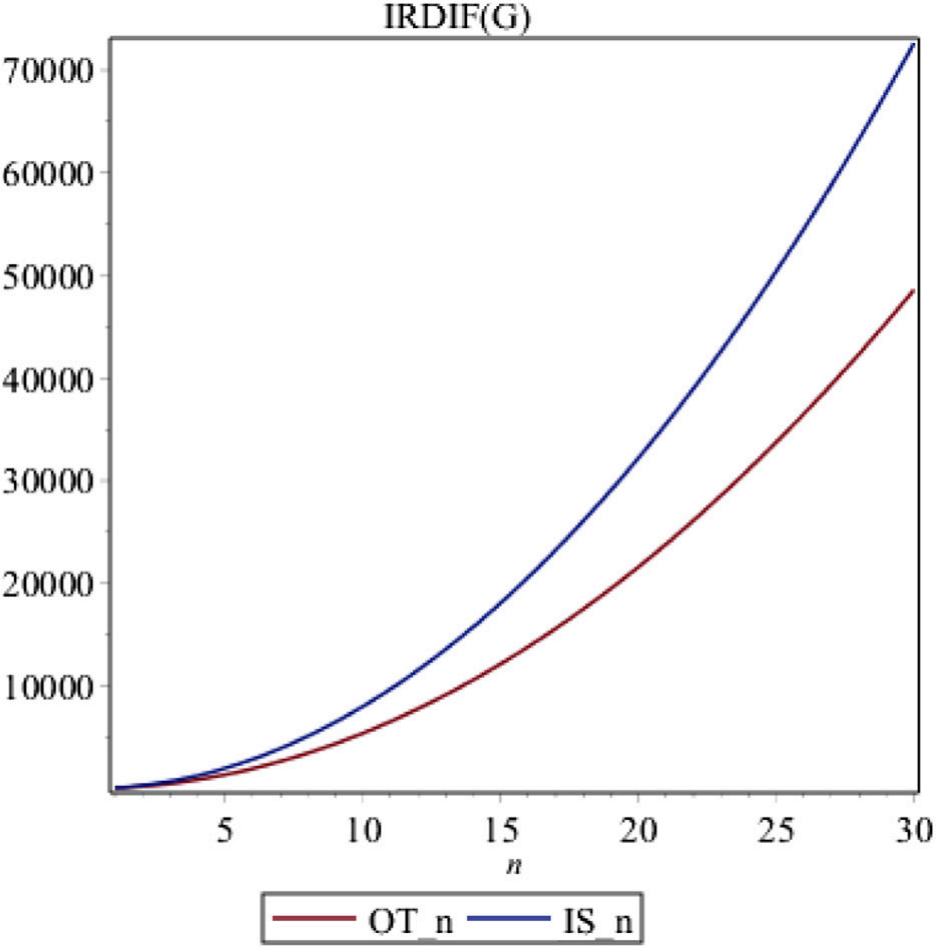
Graphs of irregularity Index *IRDIF*.

In this part, we conclude our findings of the irregularity indices for these three structures. We use red and blue colors for *OT*_*n*_ and *IS*_*n*_, respectively. From Figure 7, it is evident that *IS*_*n*_. is highly irregular than *OT*_*n*_.

Here we have analyzed the irregularity on the basis of *IRDIF*. Choosing different irregularity measure, results can vary. However, their graphs can be constructed by any software and results can be analyzed with ease. It is clear from the theorem 1 that all irregularity measures are quadratic so they increase rather quickly. Similarly, the same quadratics are obtained so we conclude that the behavior of all irregularity indices behave similarly so we have only plotted a single irregularity measure.

In this article, we investigated the irregularity measures of various octahedral structures, computing closed forms for many of these indices. The structural dependencies of these measures were analyzed through the provided graphs. These insights can be utilized to control and predict the physical and chemical properties of these networks. Additionally, the results offer a foundation for the development of new, complex networks and structure.

## Data Availability

The original contributions presented in the study are included in the article/supplementary material, further inquiries can be directed to the corresponding author.
